# Methyl Antcinate A Suppresses the Population of Cancer Stem-Like Cells in MCF7 Human Breast Cancer Cell Line

**DOI:** 10.3390/molecules18032539

**Published:** 2013-02-26

**Authors:** Chih-Yu Peng, Pin-Chung Fong, Cheng-Chia Yu, Wan-Chi Tsai, Yew-Min Tzeng, Wen-Wei Chang

**Affiliations:** 1 School of Dentistry, Chung Shan Medical University, Taichung 402, Taiwan; E-Mails: cyp@csmu.edu.tw (C.-Y.P.); ccyu@csmu.edu.tw (C.-C.Y.); 2 Department of Dentistry, Chung Shan Medical University Hospital, Taichung 402, Taiwan; 3 Department of Biomedical Sciences, Chung Shan Medical University, Taichung 402, Taiwan; E-Mail: chungfongpin@hotmail.com; 4 Institute of Oral Science, Chung Shan Medical University, Taichung 402, Taiwan; 5 Department of Medical Laboratory Science and Biotechnology, Kaohsiung Medical University, Kaohsiung 807, Taiwan; E-Mail: wanchi@kmu.edu.tw; 6 Department of Laboratory Medicine, Kaohsiung Medical University Hospital, Kaohsiung 807, Taiwan; 7 Institute of Biochemical Sciences and Technology, Chaoyang University of Technology, Taichung 413, Taiwan; E-Mail: ymtzeng@cyut.edu.tw; 8 Department of Medical Research, Chung Shan Medical University Hospital, Taichung 402, Taiwan

**Keywords:** methyl antcinate A, breast cancer stem-like cells, p53, Hsp27, IκBα

## Abstract

Methyl antcinate A (MAA) is an ergostane-type triterpenoid extracted from the fruiting bodies of *Antrodia camphorate* that has been reported to be a cytotoxic agent towards some types of cancer cells, such as oral cancer and liver cancer. Cancer stem cells (CSCs) are a particular population within cancer cells which are responsible for tumor initiation, drug resistance and metastasis and targeting CSCs is an emerging area in cancer therapy. In this study, we examine the effect of MAA on cancer stem-like cells in the MCF7 human breast cancer cell line. Although MAA displayed very low cytotoxic effect towards MCF7 under normal culture conditions, it did show good inhibitory effects on the self-renewal capability which was examined by mammosphere culture including primary and secondary sphere. MAA also inhibited cell migration ability of MCF7 sphere cells. By western blot analysis, MAA was shown to suppress the expression of heat shock protein 27 and increase the expression of IκBα and p53. In conclusion, our data demonstrate that MAA has anti-CSC activity and is worthy of future development of potent anticancer agents.

## 1. Introduction

*Antrodia camphorata* (Niu-Chang-Chih or Zhan-Ku), Polyporaceae, is a medicinal mushroom that has been widely used as a herbal medicine in Taiwan for its liver protection, anti-inflammation and anticancer properties [1]. Several biological activities of crude extract of *A. camphorata* has been examined, such as its hepatoprotective, immunomodulatory and anticancer effects [2]. Regarding the anticancer properties of *A. camphorata*, its crude extract has been reported to induce apoptosis in some cancer cells [2]. In order to avoid the discrepancy of the pharmaceutical effects of crude extract, pure effective compounds have been extracted from fruit bodies of *A. camphorata* and it was found that triterpenoids are the major representative phytoconstituents [3]. Among these triterpenoids, methyl antcinate A (MAA) belongs to the ergostane-type triterpenoids [4] and has been demonstrated to have anti-proliferation effects in some types of cancer cells. In Huh7, a human liver cancer cell line, MAA could induce apoptosis through induction of reactive oxygen species-mediated mitochondrial translocation of cofilin and the Bax-triggered mitochondrial death pathway [5]. Similar effects and mechanisms could also be observed in MAA-treated oral cancer [4] or prostate cancer [6] cells. These reports suggest that MAA is a potent anticancer agent.

Cancer stem cells (CSCs) have been discovered in a variety of solid tumors, and have been considered a particular subpopulation within cancer cells required to initiate and maintain tumors [7,8,9]. CSCs also play important roles in resistance to chemotherapy [10,11,12] and radiotherapy [13] and have been suggested to be the cells responsible for metastasis [14]. In breast cancer, CSCs could be identified as cells with CD24^−^CD44^+^ marker [15] or high intracellular aldehyde dehydrogenase activity [16]. In addition, breast CSCs could also been enriched by spheroid culture which represents their self-renewal capability *in vitro* [17]. Because of the importance of CSCs in cancer biology, to target them has been suggested to be the emerging area in the development of cancer therapy. In this study, we sought to evaluate the effect of MAA on MCF7, a human breast cancer cell line, and provide the molecular mechanism(s) for future development of modifications of such triterpenoids.

## 2. Results

### 2.1. MAA Suppressed Self-Renewal Capability of MCF7 Mammospheres

**Figure 1:**
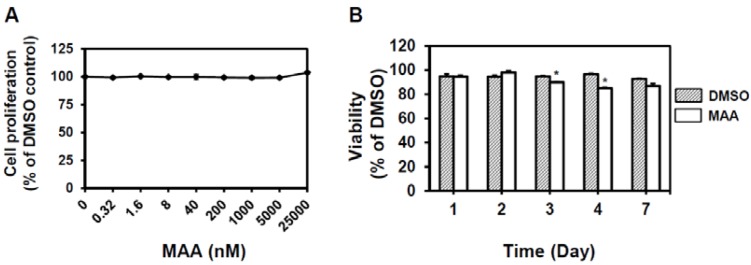
The cytotoxic effect of MAA towards MCF7 cells under normal culture conditions. (**A**) Proliferation of MCF7 at 48 h under the indicated concentration of MAA was determined as described in Experimental Section 4.2. DMSO (0.1%) was used as vehicle control and results were presented as relative percentage to DMSO. (**B**) Time course determination of viability of MCF7 cells in presence of MAA (50 μM) was examined as described in Experimental Section 4.2. *, *p* < 0.05.

We first examined the cytotoxicity of MAA towards whole populations of MCF7 cells under normal culture conditions. Up to a concentration of 25 μM, MAA displayed no cytotoxic effect on MCF7 cells after a 48 h incubation period under normal conditions (Figure 1A). We also tested the time course effect of 50 μM of MAA to cell viability of MCF7 cells under normal culture conditions and the results indicated that MAA had minor cytotoxic effects on MCF7 cells up to 72 h [down to (90 ± 0.2)% (*p* = 0.003)] or 96 h [down to (84.8 ± 0.1)% (*p* = 0.001)] (Figure 1B). Treatment of 50 μM MAA in normal cultured MCF7 cells did not significantly induce cell death at Day 7 (Figure 1B, *p* = 0.065 when compared with DMSO treated cells). Overall, MAA has a minor cytotoxic effect towards MCF7 cells under normal culture condition.

**Figure 2:**
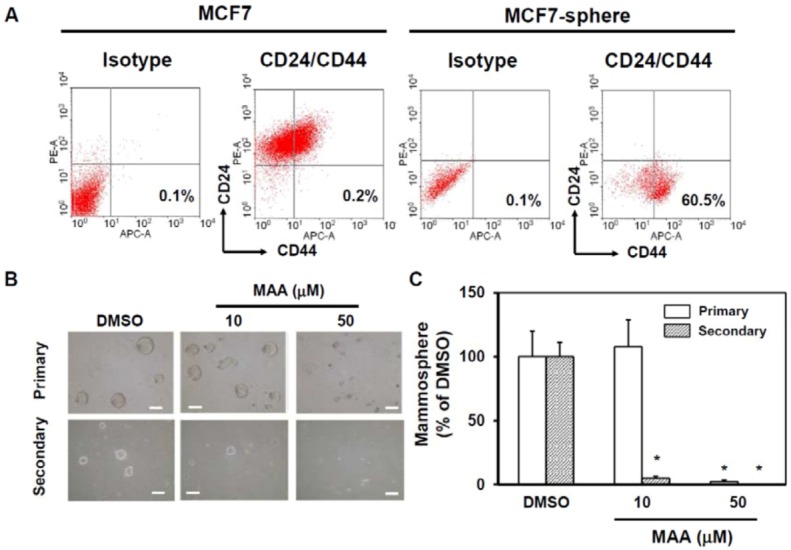
MAA displayed a suppressive effects on the self-renewal capability of CSCs within MCF7 cells. 2 × 10^4^ MCF7 cells/well were seeded into ultralow attachment 6-well-plate to form mammospheres and performed flow cytometric analysis of the expression of CD24^−^CD44^+^ BCSC marker (**A**). Primary and secondary mammosphere culture with 0.1% DMSO or indicated concentration of MAA as described in Experimental Section 4.3. Pictures of primary or secondary mammospheres were shown in (**B**) and inserted bars presented 100 μm. The counting results were shown in (**C**). *, *p* < 0.05.

To determine if MAA has any suppressive effect on the self-renewal capability of CSCs within MCF7 cells, we applied mammosphere culture and added MAA in the medium at the beginning of culture. Mammopshere culture has been used to enrich CSCs in MCF7 cells [17]. We also confirmed that MCF7 sphere cells were enriched with CD24^−^CD44^+^ BCSC marker (0.2% in normal culture condition and 60.5% in sphere cells, Figure 1A). According to mammosphere analysis, MAA could suppress the formation of mammospheres of MCF7 cells at 50 μM in both primary and secondary spheres [Figure 2B (right panel) and Figure 2C]. Although MAA did not inhibit the formation of primary spheres of MCF7 cells at 10 μM [Figure 2B (middle upper) and Figure 2C], but it displayed an inhibitory effect to the formation of secondary spheres [Figure 2B (middle lower) and Figure 2C] suggesting that MAA has a suppressive effect on mammosphere formation capability of CSCs within MCF7 at concentrations as low as 10 μM.

### 2.2. MAA Inhibited Cell Migration Ability of MCF7 Sphere Cells

**Figure 3:**
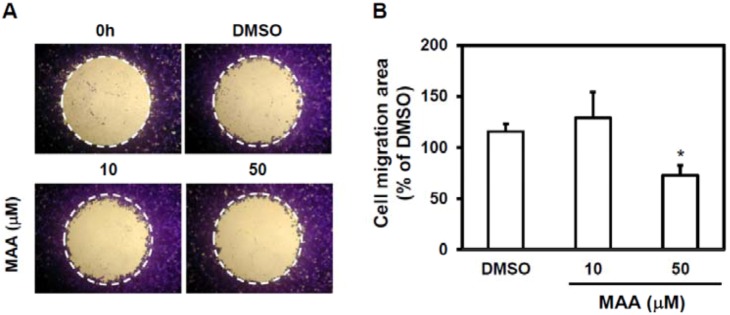
MAA inhibited cell migration ability of MCF7 sphere cells. MCF7 sphere cells were cultured from mammosphere culture for 7 d and digested into single cell suspension by acctuase. Single cell suspension of MCF7 spheres (5 × 10^4^/100 μL/well) was used for wound-healing based cell migration assay as described in Experimental Section 4.5. (**A**) Pictures of each well were taken by inverted microscopy and cells within dotted circle indicating the migrated cells. (**B**) Cell migration area were further analyzed by ImageJ software and presented as relative percentage of DMSO control. *, *p* < 0.05.

Cell migration ability is the first step of tumor metastasis [18] and CSC has been suggested to contribute to metastasis [14]. We next examined if MAA has anti-migration effect to MCF7 sphere cells. With wound-healing assay, 50 μM of MAA displayed a suppressive effect of MCF7 sphere cells (Figure 3). It suggests that MAA may also be a potent anti-metastasis agent.

### 2.3. MAA Inhibited the Expression of Heat Shock Protein 27 (Hsp27) and Increased the Expression of IκBα and p53 in MCF7 Sphere Cells

**Figure 4:**
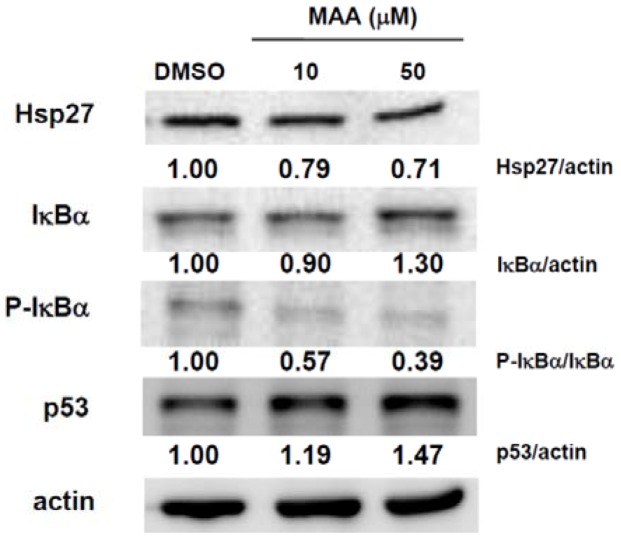
MAA decreased the expression of Hsp27 and increased the expression of IκBα and p53 in MCF7 sphere cells. MCF7 cells were cultured under mammopshere condition for 5 d, treated with 0.1% DMSO or indicated concentration of MAA for further 48 h and spheres were harvested for western blot analysis. The inserted numbers were indicated as relative expression of interested protein when compared with DMSO group.

We have previously demonstrated that Hsp27 could regulate the maintenance of breast CSCs through EMT and nuclear factor k B (NFκB) [19]. We next examined if MAA could suppress the expression of Hsp27 in MCF7 sphere cells. As shown in Figure 4, MAA inhibited Hsp27 expression in MCF7 sphere cells in a dose-dependent manner. MAA also increased IκBα expression, which is the negative regulator of NFκB, through decreased the phosphorylation of IκBα (Figure 4). It also suggests that MAA may inhibit the activation of NFκB. In addition, we also found that MAA could also increase the expression of p53 (Figure 4), which is a tumor suppressor and has been demonstrated to suppress the self-renewal capability of breast CSCs [20]. From these results, it indicates that MAA could suppress the CSC-like population of MCF7 cells through inhibition of Hsp27 and induction of IκBα and p53.

## 3. Discussion

Triterpenoids are phytochemicals which are metabolites of isopentenyl pyrophosphate oligomers and more than twenty thousand trierpenoids exist in Nature [21]. Several triterpenoids have been demonstrated to have cytotoxicity to cancer cells and anti-tumor effect in animal model of cancer [22], but information about the anti-CSC effects of triterpenoids is limited. Our study of MAA indicated that MAA affects some CSC features in MCF7 breast cancer cells including cell migration and mammosphere formation capability. Recently, Lup-20(29)-en-3β-ol (lupeol), a triterpenoid found in fruits and vegetables, has been demonstrated to target tumor-initiating cells (TICs, another term referring to CSCs) of liver cancer [23]. Lupeol could suppress hepatic sphere formation of hepatocellular carcinoma (HCC) cells, inhibit tumorigenicity of HCC TICs in nude mice and sensitize HCC TICs to chemotherapy agents [23]. Knockdown of phosphatase and tensin homolog (PTEN) could abolish the effect of lupeol to HCC TICs which demonstrated that the effects of lupeol in HCC TICs were mediated by PTEN [23]. Our study of MAA in targeting breast CSCs revealed that MAA could suppress self-renewal of CSC-like cells in MCF7 partly through up-regulation of p53. It will be suggested that testing for the triterpenoids which could elevate p53 expression may be a strategy for searching CSC targeting triterpenoids. It has been shown that uncarinic acids, the triterpenoids isolated from *Uncaria rhynchophylla*, could inhibit cell proliferation of MCF7 cells which was associated with the up-regulation of p21/WAF1 and inhibition of cyclinB1, cyclinA, Cdc2, and Cdc25C in a p53-dependent manner [24]. It will be interesting to test if these uncarinic acids also have anti-CSC activity.

Hsps have been reported to be overexpressed in cancers and function as cytoprotection proteins in against apoptosis induced by chemotherapy or radiation therapy [25]. Among the Hsp family, the expression of Hsp27 has been reported to be associated with poor prognosis in gastric, liver and prostate carcinoma [26]. Inhibition of Hsp27 with a small molecule inhibitor decreased the invasion capability of metastatic human breast cancer cells [27] indicating that Hsp27 may regulate the metastasis of breast cancer. Here we report that MAA could suppress the expression of Hsp27 in breast CSC-like cells and lead to inhibit their self-renewal capability. It has been reported that avicins, plant triterpenoids, could induce the degradation of Hsp70 and lead to the induction of apoptosis in leukemia cells [28]. Celastrol, a quinonemethide triterpene derived from the medicinal plant *Tripterygium wilfordii*, could function as an ATP-independent inhibitor of Hsp90 to induce apoptosis of pancreatic cancer cells [29]. We have previously demonstrated that Hsp27 regulates EMT features and NF-κB activity in BCSCs [19]. Knockdown of Hsp27 inhibited the tumorigenicity of BCSCs [19]. Hsp27 also plays a role in the chemoresistance feature of lung CSCs [30]. Blockage of Hsp27 with a small molecule inhibitor or a plant flavonoid decreased the survival of lung CSCs when combined with chemotherapy [30]. It will be interesting to investigate how MAA inhibit Hsp27 such as to test its effect to heat shock factors, the transcriptional regulators of Hsps.

Our study also indicated that MAA might inhibit NFκB activity since it could up-regulate IκBα expression. It has been reported that celastrol could suppress NFκB activation and led to potentiate tumor cells toward tumor necrosis factor induced apoptosis [31]. Suppression of NFκB activation by celastrol also led to the inhibition of the invasive capability of tumor cells [31]. In addition to natural triterpenoids, a large number of synthetic triterpenoids have been synthesized by structural modification from natural triperpenoids to optimize their bioactivities [21]. It has been reported that CDDO-Me, a synthetic triterpenoid, could inhibit NFκB activation in human leukemia cell lines and led to down-regulate of NFκB target genes which involved in anti-apoptosis, proliferation or angiogenesis [32]. It is worthy to develop synthetic triterpenoids based on the structure of MAA and further screen of more potent derivatives with anti-CSC activity in the future.

## 4. Experimental

### 4.1. Preparation of MAA

MAA was purified from the fruiting bodies of *A. camphorata* as described in [33]. Briefly, the air-dried powder of the fruiting bodies was extracted with *n*-hexane, chloroform, and methanol under reflux. After exhaustive extraction and concentration, the chloroform soluble fraction was chromatographed over silica gel using n-hexane/ethyl acetate gradient eluent, and produced six fractions. The sixth fraction was purified by silica gel column chromatography using a chloroform/methanol gradient from 100% chloroform to 20% methanol to yield antcin A and a mixture of compounds that was further separated by column chromatography using chloroform/methanol elution to obtain MAA.

### 4.2. Cell Culture, Proliferation and Viability Assay

MCF7 cells was obtained from American Type Culture Collection (Manassas, VA, USA) and cultured in MEMα medium (Invitrogen Corporation, Grand Island, NY, USA) containing 10% fetal bovine serum (Biological Industries, Kibbutz Beit-Haemek, Israel) and 5 μg/mL insulin (Sigma-Aldrich, St. Louis, MO, USA). For cell proliferation assay, MCF7 cells were seeded as 2 × 10^4^ cells/well in 96-well-plate with 0.1% DMSO or different concentration of MAA for 48h. Cell proliferation were determined by WST-1 cell viability assay reagent (BioVision, Mountain View, CA, USA) using a microplate reader (Molecular Devices, Sunnyvale, CA, USA) according to the manufacturer’s recommendations. The absorbance values at 440 nm of DMSO control were set as 100% of cell proliferation. For time course experiments, cell viability was determined by counting with trypan blue stain and data were presented as relative percentage of DMSO.

### 4.3. Mammosphere Assay

MCF7 cells were prepared as density of 1 × 10^4^ cells/mL in DMEM/F12 medium (Invitrogen Corporation) contain 0.5% methylcellulose (Sigma-Aldrich), 0.4% bovine serum albumin (Sigma-Aldrich), 10 ng/mL of EGF (PeproTech, Rocky Hill, NJ, USA ), 10 ng/mL bFGF (PeproTech), 5 μg/mL insulin, 1 μM hydrocortisone (Sigma-Aldrich) and 4 μg/mL heparin (Sigma-Aldrich). Cell suspension (2 mL) was seeded into wells of ultralow attachment 6-well-plate (Corning, Lowell, MA, USA) and incubated for 7 d. For secondary spheres, the cells were collected from accutase (Merck Millipore, Billerica, MA, USA) treated primary spheres, seeded as density of 2,500 cells/mL and cultivated for further 7 d.

### 4.4. Flow Cytometric Analysis of CD24 and CD44 Expression

Anti-human CD24-PE and anti-human CD44-APC conjugated antibodies were purchased from BD Biosciences (San Jose, CA, USA). 1 × 10^5^ cells were suspended in 100 μL PBS/0.01% NaN_3_ and stained with 0.1 μg fluorescent-conjugated antibodies on ice for 30 min. After washing with 2 mL PBS/0.01% NaN_3_, stained cells were analyzed with Epics XL flow cytometer (Beckman Coulter Inc., Brea, CA, USA). Data were further analyzed with WinMDI software (National Institutes of Health, Bethesda, MD, USA).

### 4.5. Cell Migration Assay

Cell migration assay was conducted by Oris Universal Cell Migration Assembly kit (Platypus Technologies, LCC, Madison, WI, USA) following manufacture’s protocol. Briefly, single cell suspension (5 × 10^4^ cells/well/100 μL) was loaded into stopper loaded wells and incubated overnight to permit cell attachment. To start cell migration, the stoppers was removed, wash wells gently with PBS, added complete cell culture medium and incubate for 16–18 h. Pictures of wells were captured with inverted microscopy after fixation and stain with 0.5% crystal violet (Sigma-Aldrich)/50% EtOH. Data were analyzed with ImageJ software (National Institute of Health, Bethesda, MA, USA).

### 4.6. Western Blot

Cells were lysed with NP-40 lysis buffer and 25 μg of total protein were separated by SDS-PAGE and transferred to PVDF membrane (Pall Corporation, Port Washington, NY, USA). Protein detection was conducted by SignalBoost^TM^ Immunodetection Enhancer kit (Merck, Darmstadt, Germany) according to manufacturer’s recommendation and signals were captured by ImageQuant LAS 4000 mini digital imaging system (GE Healthcare Life Science, Piscataway, NJ, USA). Hsp27 antibody was purchased from Stressgen (Ann Arbor, MI, USA). IκBα and phosphor-IκBα antibodies were purchase from Cell Signaling Technology (Danvers, MA, USA) p53 and actin antibodies were purchased from Santa Cruz Biotechnology, Inc. (Santa Cruz, CA, USA).

### 4.7. Statistical Analysis

Results of different experimental groups were analyzed by Student’s *t-*test and *p* values < 0.05 were considered statistically significant.

## 5. Conclusions

In conclusion, our data indicates that MAA, an ergostane-type triterpenoid extracted from the fruiting bodies of *A. camphorate* has anti-breast CSC activity and the effects are mediated by the inhibition of Hsp27 expression and increased expression of p53 and IκBα. MAA is worthy for further development of a CSC targeting agent in breast cancer therapy.
